# Supplementary material for: Response-Related Factors of Bone Marrow-Derived Mesenchymal Stem Cells Transplantation in Patients with Alcoholic Cirrhosis
Source: J Clin Med. 2019 Jun 17;8(6):862. doi: 10.3390/jcm8060862 (PMC6616969; doi:10.3390/jcm8060862)
Supplement: Supplementary file 1 [file jcm-08-00862-s001.docx]

**Supplementary files**

**Supplement table 1. Multivariate analysis of predictive factors for the responder**

| **Variables** | ***p*-value** | **Odds ratio** |
| --- | --- | --- |
| Age | NS |  |
| Gender (male/female*) | NS |  |
| Child-Pugh score | NS |  |
| MELD score | NS |  |
| AST | NS |  |
| ALT | NS |  |
| ALP | NS |  |
| High laennec score | 0.007 | 3.73 |
| Treatment with BM-MSCs (yea/no*) | 0.033 | 5.75 |

* reference category

MELD, model for end stage liver disease; AST, aspartate aminotransferase; ALT, alanine aminotransferase; ALP, alkaline phosphatase; BM-MSC, bone marrow-derived mesenchymal stem cell

**Supplement table 2. Significant cellular process between alcoholic cirrhosis and control group**

| **Term** | **P-value** | **Genes** |
| --- | --- | --- |
| Cell part | 0.008 | RASA4B, GFRA1, INHBB, KIR3DL2, P4HA1, PDK1, SERPINF1, BNIP3, STC1, ZFY |
| Cell | 0.008 | RASA4B, GFRA1, INHBB, KIR3DL2, P4HA1, PDK1, SERPINF1, BNIP3, STC1, ZFY |
| Extracellular region part | 0.013 | FBN2, GFRA1, INHBB, SERPINF1, STC1 |
| Extracellular region | 0.024 | FBN2, GFRA1, INHBB, SERPINF1, STC1 |
| Oxidoreductase complex | 0.024 | P4HA1, PDK1 |
| Extracellular matrix component | 0.033 | FBN2, SERPINF1 |
| Cytoplasm | 0.04 | RASA4B, INHBB, P4HA1, PDK1, SERPINF1, BNIP3, STC1 |
| Cytoplasmic part | 0.043 | RASA4B, INHBB, P4HA1, PDK1, SERPINF1, BNIP3 |
| Extracellular space | 0.046 | INHBB, SERPINF1, STC1 |

RASA4B, RAS P21 protein activator 4B; GFRA1, GDNF family receptor alpha-1; INHBB, inhibin subunit beta B; KIR3DL2, killer cell immunoglobulin like receptor, three Ig domains and long cytoplasmic tail 2; P4HA1, prolyl 4-hydroxylase subunit alpha-1; PDK1, pyruvate dehydrogenase kinase 1; SERPINF1, pigment epithelium-derived factor (PEDF); BNIP3, BCL2 interacting protein 3; STC1, Stanniocalcin-1; ZFY, zinc finger protein Y-linked; FBN2, fibrillin-2 precursor

**Supplement table 3. Top 10 biological process between alcoholic cirrhosis and control group**

| **Term** | **P-value** | **Genes** |
| --- | --- | --- |
| Response to abiotic stimulus | 0.0002 | INHBB, PDK1, SERPINF1, BNIP3, STC1 |
| Response to external stimulus | 0.0003 | GFRA1, INHBB, PDK1, SERPINF1, BNIP3, STC1 |
| Regulation of biological process | 0.0003 | RASA4B, FBN2, GFRA1, INHBB, KIR3DL2, PDK1, SERPINF1, BNIP3, STC1, ZFY |
| Response to stimulus | 0.0004 | RASA4B, FBN2, GFRA1, INHBB, KIR3DL2, PDK1, SERPINF1, BNIP3, STC1 |
| Response to chemical | 0.0004 | FBN2, GFRA1, INHBB, PDK1, SERPINF1, BNIP3, STC1 |
| Cellular response to chemical stimulus | 0.0004 | FBN2, INHBB, PDK1, SERPINF1, BNIP3, STC1 |
| Cellular response to hypoxia | 0.0005 | PDK1, BNIP3, STC1 |
| Cellular response to decreased oxygen | 0.0005 | PDK1, BNIP3, STC1 |
| Biological regulation | 0.0005 | RASA4B, FBN2, GFRA1, INHBB, KIR3DL2, PDK1, SERPINF1, BNIP3, STC1, ZFY |
| Cellular response to oxygen levels | 0.001 | PDK1, BNIP3, STC1 |

INHBB, inhibin subunit beta B; PDK1, pyruvate dehydrogenase kinase 1; SERPINF1, pigment epithelium-derived factor (PEDF); STC1, stanniocalcin-1; BNIP3, BCL2 Interacting protein 3; GFRA1, GDNF family receptor alpha-1; RASA4B, RAS P21 protein activator 4B; FBN2, fibrillin-2 precursor; KIR3DL2, killer cell immunoglobulin like receptor, three Ig domains and long cytoplasmic tail 2; ZFY, zinc finger protein, Y-linked

**Supplement table 4. Significant molecular function between alcoholic cirrhosis and control group**

| **Term** | **P-value** | **Genes** |
| --- | --- | --- |
| Binding | 0.003 | RASA4B, FBN2, GFRA1, INHBB, P4HA1, PDK1, SERPINF1, BNIP3, STC1, ZFY |
| Protein Binding | 0.012 | FBN2, GFRA1, INHBB, P4HA1, PDK1, SERPINF1, BNIP3, STC1 |
| Hormone Activity | 0.031 | INHBB, STC1 |

INHBB, inhibin subunit beta B; PDK1, pyruvate dehydrogenase kinase 1; SERPINF1, pigment epithelium-derived factor (PEDF); BNIP3, BCL2 interacting protein 3; GFRA1, GDNF family receptor alpha-1; RASA4B, RAS P21 protein activator 4B; FBN2, fibrillin-2 precursor; ZFY, zinc finger protein, Y-Linked; STC1, stanniocalcin-1; P4HA1, prolyl 4-hydroxylase subunit alpha-1.

**Supplement table 5. Profiling of up or down regulated gene expression in BM derived MSCs between alcoholic cirrhosis and control group**

| Gene | Fold Change | Function |
| --- | --- | --- |
| GFRA1 | 2.08 | Cell development, control of neuron survival and differentiation. |
| FBN2 | 2.05 | Regulation of transforming growth factor beta receptor signaling pathway |
| RASA4B | 1.61 | Regulate cell growth and differentiation, Regulating cell proliferation, survival, growth, migration and differentiation. |
| KIR3DL2 | 1.61 | Regulation of immune system process, cellular defense response |
| MIR4436A | 1.59 | NA |
| SERPINF1 | 1.56 | Regulation of stem cell proliferation, supporting stem cell survival and maintaining multipotency.  Release by resident stem cells that stimulate migration of cells in early regeneration. |
| STC1 | -2.25 | Cell morphogenesis involve in differentiation, multi cellular organismal development, negative regulation of cell migration.  Inhibition of ROS production/reduction of ER stress/diminishing TGF beta . |
| RNU5F-1 | -2.23 | NA |
| TEKT4P2 | -1.96 | NA |
| ZFY | -1.69 | Regulation of biosynthetic process, regulation of cellular process. |
| BNIP3 | -1.63 | Immune system process, signal transduction, cell differentiation, negative regulation of apoptotic process. |
| LOC105376694 | -1.62 | NA |
| PDK1 | -1.60 | Biosynthetic process, stem cell proliferation and migration. |
| INHBB | -1.58 | Transforming growth factor beta receptor binding, growth factor activity, protein secretion. |
| P4HA1 | -1.50 | Key enzyme in collagen synthesis, collagen fibril organization. |

GFRA1, GDNF family receptor alpha-1; FBN2, fibrillin-2 precursor; RASA4B, RAS P21 protein activator 4B; KIR3DL2, killer cell immunoglobulin like receptor, three Ig domains and long cytoplasmic tail 2; MIR4436A, microRNA 4436a; SERPINF1, pigment epithelium-derived factor (PEDF); STC1, stanniocalcin-1; RNU5F-1, RNA, U5F small nuclear 1; TEKT4P2, tektin 4 pseudogene 2; ZFY, zinc finger protein, Y-Linked; BNIP3, BCL2 interacting protein 3; PDK1, pyruvate dehydrogenase kinase 1; INHBB, inhibin subunit beta B; P4HA1, prolyl 4-hydroxylase subunit alpha 1
